# A Case of Burnt-Out Cardiac Sarcoidosis Presenting With Sustained Ventricular Tachycardia

**DOI:** 10.7759/cureus.28931

**Published:** 2022-09-08

**Authors:** Sherin Sallam, Zaid Shahrori, Mariam Rana, Claire Sullivan

**Affiliations:** 1 Department of Medicine, Case Western Reserve University/University Hospitals, Cleveland, USA; 2 Department of Medicine, Hashemite University, Zarqa, JOR; 3 Department of Cardiology, Case Western Reserve University/University Hospitals, Cleveland, USA

**Keywords:** sarcoidosis, f-fluorodeoxyglucose positron emission tomography - computed tomography, cardiac magnetic resonance (cmr), multimodality cardiac imaging, ventricular tachycardia (vt)

## Abstract

Cardiac sarcoidosis is a challenging clinical entity in terms of diagnosis and management. Cardiac involvement is the most common cause of death in patients with sarcoidosis. Recently, there have been new advancements in the imaging modalities that aid in the diagnosis of this condition, including cardiac MRI and PET scan. These tools can help identify and determine the extent of the progression of sarcoidosis, which can have diagnostic and therapeutic implications. In this report, we present the case of a 74-year-old man with no history of sarcoidosis who presented with sustained ventricular tachycardia (VT) and was subsequently found to have findings consistent with burnt-out sarcoidosis on imaging. This case highlights the differences in the management of various stages of cardiac sarcoid involvement to reduce adverse outcomes.

## Introduction

Sarcoidosis is a challenging clinical entity that is characterized by granuloma formation in multiple organs with multiple postulated triggers that have yet to be confirmed. It usually involves multiple organs, the lungs being the most commonly affected organs followed by the skin, liver, and gastrointestinal tract [1]. Cardiac involvement is clinically diagnosed in as few as 5% of patients with sarcoidosis; on the other hand, some studies have shown evidence of disease in the myocardium in up to 25% of autopsies of the said population [2]. The definitive diagnosis of sarcoidosis requires histopathological evidence of the classic “non-caseating granulomas”, which can be obtained via biopsy from any radiographically or clinically accessible lesion. However, due to the heterogeneous nature of granulomatous involvement, cardiac sarcoidosis cannot be ruled out if an endomyocardial biopsy (EMBx) is negative [3].

Recently, advancements in cardiac imaging like cardiac MRI (cMRI) and F-fluorodeoxyglucose PET-CT (FDG PET/CT) have enabled a probabilistic approach to characterize cardiac sarcoidosis. Specifically, the use of hybrid or fusion imaging techniques or side-by-side comparison of the images can help indicate the stage of the disease [4]. We discuss the case of a 74-year-old man who presented with sustained ventricular tachycardia (VT) and was found to have imaging findings consistent with “burnt-out” cardiac sarcoidosis. This characterization had significant management implications for the patient in this case.

## Case presentation

A 74-year-old African-American man with a past medical history most notable for mild aortic stenosis, hypertension, and rheumatic fever in childhood presented to the emergency department complaining of lightheadedness for five days. The patient stated that he had been having occasional episodes of lightheadedness associated with exertion that had resolved with rest over the past few years. The episodes were also associated with shortness of breath and fatigue and had worsened five days prior to the presentation, prompting him to seek medical care. He denied chest pain, palpitations, or lower extremity swelling.

**Figure 1:**
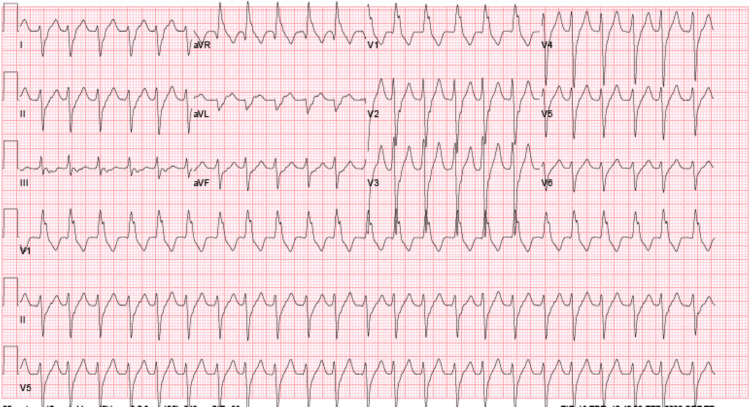
ECG showing a rate of 140 bpm, a wide QRS complex, and right bundle branch block morphology suggestive of monomorphic ventricular tachycardia ECG: electrocardiogram

In the emergency department, initial vital signs revealed a temperature of 36.2 °C, heart rate of 117 beats per minute, blood pressure of 108/68 mmHg, respiratory rate of 18 breaths per minute, and oxygen saturation of 99% on ambient air. Physical examination was notable for tachycardia, a soft systolic ejection murmur, absence of jugular venous distension or lower extremity edema, clear air entry bilaterally in the lung fields, and absence of distension or tenderness in the abdomen. An electrocardiogram showed a rate of 140 bpm, with a wide QRS complex, with right bundle branch block (RBBB) morphology suggestive of monomorphic VT (Figure 1). Initial complete blood count was unremarkable for leukocytosis and notable for a hemoglobin level of 10.5 g/dL consistent with the patient’s known baseline. The renal panel was notable for a potassium level of 3.6 mEq/L and a magnesium level of 1.6 mg/dL with a creatinine level of 0.9 mg/dL. High-sensitivity troponin was measured at 23.8 ng/L.

Telemetry monitoring showed sustained monomorphic VT at a rate of 140 bpm. Subsequently, 150 mg of amiodarone was administered followed by a continuous infusion along with 4 g of magnesium with subsequent conversion to normal sinus rhythm. The patient was admitted for further diagnostic testing and management. Upon further questioning, he denied the use of any QT-prolonging medications or personal or family history of cardiac arrhythmia.

Transthoracic echocardiography was performed and showed a left ventricular ejection fraction (LVEF) of 60-65%, mild concentric left ventricular hypertrophy, mildly dilated left atrium, mild aortic stenosis, and mild to moderate diastolic dysfunction. Coronary angiography was subsequently performed and showed non-obstructive coronary artery disease (CAD) and elevated left ventricular end-diastolic pressure (LVEDP) at 19 mmHg. cMRI was pursued and showed LVEF of 56%, no regional wall motion abnormalities, and no evidence of myocardial edema/inflammation on T2. Mid-wall fibrosis of the basal/apical septum and the basal lateral wall was evident on late gadolinium enhancement (LGE) imaging. A PET scan did not show any evidence of inflammation or finding suggestive of malignancy. There was minimal to borderline activity in the bilateral axillary lymph nodes, along with a sub-centimeter moderate focus of activity in the left adrenal gland.

The patient underwent successful implantation of a dual chamber implantable cardioverter-defibrillator (ICD). During the hospitalization, he was maintained on oral amiodarone and started on metoprolol tartrate with the maintenance of normal sinus rhythm. He was discharged home on oral amiodarone and metoprolol tartrate 25 mg. At the follow-up visit one month later, no ICD shocks were identified, and he is scheduled for an EMBx for further diagnostic testing.

## Discussion

We discussed the case of a 74-year-old African-American man who presented with sustained monomorphic VT in the absence of clinically significant CAD. On cMRI, he was found to have mid-wall fibrosis of the basal/apical septum, and the basal lateral wall was evident on LGE imaging, without any evidence of inflammation on FDG PET-CT. This picture was overall consistent with “burnt-out” cardiac sarcoidosis given the absence of active inflammation on PET imaging, with evidence of fibrosis on cMRI. Identifying the specific stage of sarcoidosis can have important diagnostic and therapeutic implications and is usually achieved by a side-by-side comparison of the imaging modalities used.

Although the heart is one of the least commonly clinically affected organs in sarcoidosis, the most common cause of death in patients with sarcoidosis is cardiac involvement with atrioventricular block, ventricular tachyarrhythmias, and heart failure [5]. VT as the initial presentation of sarcoidosis is exceptionally rare. In a case report by Mori et al., a patient presented with sudden cardiac arrest due to persistent VT, with autopsy results later showing evidence of cardiac sarcoidosis [6]. In another study, a 41-year-old lady presented with VT and was found to have systemic sarcoidosis with cardiac involvement; she was started on steroids, amiodarone, and metoprolol, and received failed radiofrequency ablation. Unfortunately, the patient passed away due to recurrent VT [7]. Therefore, maintaining a high index of early suspicion of sarcoidosis in patients presenting with arrhythmias, especially in the absence of CAD, is critical to prevent disease-related mortality.

To our knowledge, this is the only reported case of “burnt-out” cardiac sarcoidosis resulting in VT. The implication of this is that in classic cardiac sarcoidosis cases, steroids and immunomodulatory agents are often promptly offered to counter the inflammation. Moreover, it has been shown that steroids have a potential benefit in patients with mild to moderately reduced ejection fraction (30-50%), but not in those with a preserved ejection fraction like our patient, or those with a severely reduced ejection fraction [8]. However, in the absence of inflammation, these agents have no proposed role, which is why they were not offered to the patient in this case. The patient was offered an ICD to prevent sudden cardiac death along with amiodarone as an antiarrhythmic agent to maintain sinus rhythm. Although the patient is scheduled to undergo EMBx, the absence of granulomas or fibrosis on biopsy will not exclude the diagnosis, but their presence will further confirm it.

## Conclusions

Cardiac sarcoidosis is a challenging clinical entity to diagnose and manage. The combined use of cMRI and FDG PET/CT can help characterize disease stage and risk-stratify the patients. Moreover, prompt device implantation for patients presenting with ventricular arrhythmias is crucial to prevent sudden cardiac death. Further research is needed to identify patients with sarcoidosis who require workup for cardiac sarcoidosis to prevent associated morbidity and mortality.
